# Increased serum levels of sortilin are associated with depression and correlated with BDNF and VEGF

**DOI:** 10.1038/tp.2015.167

**Published:** 2015-11-10

**Authors:** H N Buttenschøn, D Demontis, M Kaas, B Elfving, S Mølgaard, C Gustafsen, L Kaerlev, C M Petersen, A D Børglum, O Mors, S Glerup

**Affiliations:** 1 Translational Neuropsychiatry Unit, Department of Clinical Medicine, Aarhus University, Aarhus, Denmark; 2 The Lundbeck Foundation Initiative for Integrative Psychiatric Research, Aarhus, Denmark; 3 Department of Biomedicine and Centre for Integrative Sequencing, Aarhus University, Aarhus, Denmark; 4 The Lundbeck Foundation Research Center, MIND, Aarhus University, Aarhus, Denmark; 5 Research Unit of Clinical Epidemiology, Institute of Clinical Research, University of Southern Denmark, Odense, Denmark; 6 Center for Clinical Epidemiology, Odense University Hospital, Odense, Denmark; 7 Research Department P, Aarhus University Hospital, Aarhus, Denmark

## Abstract

Neurotrophic factors have been investigated in relation to depression. The aim of the present study was to widen this focus to sortilin, a receptor involved in neurotrophic signalling. The serum sortilin level was investigated in 152 individuals with depression and 216 control individuals, and eight genetic markers located within the *SORT1* gene were successfully analysed for association with depression. Genotyping was performed using the Sequenom MassARRAY platform. All the individuals returned a questionnaire and participated in a semi-structured diagnostic interview. Sortilin levels were measured by immunoassay, and potential determinants of the serum sortilin level were assessed by generalized linear models. Serum levels of brain-derived neurotrophic factor (BDNF) and vascular endothelial growth factor (VEGF) were measured in previous studies. We identified a significant increase of serum sortilin levels in depressed individuals compared with controls (*P*=0.0002) and significant positive correlation between serum sortilin levels and the corresponding levels of BDNF and VEGF. None of the genotyped SNPs were associated with depression. Additional analyses showed that the serum sortilin level was influenced by several other factors. Alcohol intake and body mass index, as well as depression, serum BDNF and serum VEGF were identified as predictors of serum sortilin levels in our final multivariate model. In conclusion, the results suggest a role of circulating sortilin in depression which may relate to altered activity of neurotrophic factors.

## Introduction

Depression is a common complex mental disorder and a worldwide leading cause of disability.^1^ The aetiology of depression is not fully understood, but it has been found to be moderately heritable.^2^ The identification of peripheral biomarkers for depression is obviously of great clinical relevance and has the potential to improve diagnosis, treatment and prognosis. Several hypothesis-driven efforts have been made to identify trait and state biomarkers, and the search for peripheral biomarkers of depression has mainly been driven by the monoaminergic, the immune-inflammatory and the neuroendocrine hypothesis.^3^ Since the first paper reporting differences in serum brain-derived neurotrophic factor (BDNF) levels between depressed individuals and healthy controls,^4^ the hypothesis of a dysregulation in the neurotrophin system in depression has also been investigated. Although neurotrophic factors remain exciting peripheral biomarker candidates, we find it relevant to widen the focus to their receptors as well.

Sortilins, also known as the Vsp10-domain receptors are unique sorting and signalling receptors expressed in the nervous system. This receptor family includes five receptors: sortilin, SorLA and SorCS1-3.^5^ Members of the sortilin family have been identified as potential neuronal disease genes, for example, *SORL1* has been associated with Alzheimer's disease^6^ and *SORCS2* with bipolar disorder and attention deficit/hyperactivity disorder.^7, 8, 9^ Sortilins binds a number of ligands through the 700 amino acid extracellular Vsp10-domain.^10, 11^ The majority of known sortilin binding partners are related to lipid metabolism and to neurotrophic factor signalling as reviewed by Glerup *et al.*^5^ Sortilin is encoded by the *SORT1* gene located on chromosome 1p13.3 (ref. 11) and has an important role in regulating BDNF activity.^12, 13, 14^ It has long been known that sortilin can form a complex with the p75 neurotrophin receptor (p75NTR) and proBDNF to induce apoptosis,^12^ and likewise, a more recent study suggests that sortilin and p75NTR are involved in trophic signalling by mature neurotrophins.^14^ The extracellular domain of sortilin (soluble sortilin) is released in both a constitutive and regulated manner by proteolysis from the surface of neuronal and other cell types.^13, 15, 16^ The function of soluble sortilin is unclear but it has been found to inhibit the conversion of proBDNF into mature BDNF by plasmin and to protect neurons from the apoptotic properties of proBDNF.^12^ Furthermore, a recent report describes that released soluble sortilin forms a complex with the BDNF receptor TrkB and the EGF receptor and that this ternary complex is internalized and subsequently released again in exosomes.^17^ Soluble sortilin can be detected in serum^18, 19^ and plasma,^13^ and has been found to correlate well with sortilin mRNA levels in the brain.^20^ The source of circulating sortilin is unclear, but it is expressed by most tissues including blood cells and platelets.^5, 13^

On the basis of the above, we speculated that sortilin has a role in depression. Thus, we investigated genetic variants located within the *SORT1* gene and measured serum sortilin levels in 152 cases with depression and 216 control individuals. We also investigated the correlation between serum sortilin levels and BDNF in addition to the correlation between serum sortilin and vascular endothelial growth factor A (VEGF) levels, another neurotrophic factor suggested to have a role in the pathophysiology of depression.^21^ A secondary aim of the study was to identify determinants influencing the serum sortilin level and to build a multivariate model.

## Materials and methods

### Population

One-hundred and fifty-two individuals with depression according to the ICD-10 diagnostic criteria for research and 216 screened control individuals were included in the study. The subjects were all part of the Danish PRISME study^22, 23^ where they returned a completed questionnaire, and participated in a semi-structured diagnostic interview.^24^ Details on the study sample are presented elsewhere.^25, 26^ The following socio-demographic variables, lifestyle indicators, and health indicators were obtained from the questionnaire: smoking (current smoker/previous smoker/never smoker), antidepressant treatment (yes/no), previous depressive episodes (yes/no), previous diagnosis of other psychiatric disorders (yes/no), physical activity (⩽/>4 h per week) and alcohol intake (female: ⩽/>20 g per day and male: ⩽/>30 g per day). Age and gender were derived from the personal identification number assigned to all live-born children and stored in the Danish Civil Registration System.^27^ Body mass index (BMI) was calculated from the measured height and weight obtained at the clinical interview. A Danish translation of a brief version of the List of Threatening Experiences Questionnaire^28, 29^ was used to record nine stressful life events up to 6 months before completion of the questionnaire. Each stressful life event was rated on a four-point scale severity with a rating of not present, mild, moderate and severe. In this analysis, only moderate and severe events were counted. Although the original List of Threatening Experiences Questionnaire consists of 12 events, we only included nine events. Two questions from the original List of Threatening Experiences Questionnaire were combined into a single item (these were: ‘did you have a separation due to marital difficulties?' and ‘did you break off a steady relationship?' which were combined into ‘did you have a separation due to marital difficulties or break off a steady relationship?'). Two questions in relation to unemployment were excluded from the present study as all the participants were either municipal or hospital employees within large public service workplaces in a Danish county. Case–control status (diagnosis of depression) was obtained from the clinical interview according to the ICD-10 diagnostic criteria.^30^

All cases and controls gave written informed consent and the study was approved by the Danish Data Protection Agency (2006-41-7032) and the Regional Ethics Committee (RRS 2006-1028 (2747-06)) in Denmark.

### Serum measurements

Blood for serum samples was collected in anticoagulant-free tubes (Terumo, Venosafe, VF-109SP, Prague, Czech Republic) between 0900 h and 1500 h and centrifuged (1550 *g*, 10 min, 4 °C) within 24 h. The supernatant was stored in aliquots at −80 °C. Serum sortilin levels were measured blinded for case and control status by sortilin-specific enzyme-linked immunosorbent assay as previously described in Gustafsen *et al.*^18^ This assay speficially detects sortilin and not the related receptors SorCS2 and SorLA at a sensitivity of ~0.1 ng ml^−1^ as shown in Supplementary Figure 1. In brief, 96-well plates (Nunc Maxisorp, Thermo Scientific, Roskilde, Denmark) were coated with 10 μg ml^−1^ rabbit anti-sortilin (5438) diluted in 100 mM NaHCO_3_, pH 9.8. Wells were blocked in 2% bovine serum albumin and samples were diluted in phosphate-buffered saline containing 0.05% Tween20 and 1% bovine serum albumin. Purified sortilin extracellular domain was used for standard dilution series.^31^ Mouse anti-human sortilin monoclonal F11 was used for detection (1 μg ml^−1^) followed by HRP-conjugated rabbit anti-mouse secondary antibodies (1:2000, P0260, Dako, Glostrup, Denmark). For additional information on the use of these antibodies, please see the online database www.pabmabs.com. Serum levels of BDNF and VEGF were measured previously in the same individuals using immunoassays from R&D Systems (Minneapolis, MN, USA) and the determination was processed according to the manufacturer's specifications.^25, 26^ The same batch numbers was used for the entire experiments and the standard curves and the samples were run in duplicates. The absorbances were measured at 450 nm with wavelength correction set to 540 nm (EL 800 Universal Microplate Reader, Bio-Tek Instruments, Winooski, VT, USA). Serum samples for VEGF measurements were analysed undiluted to be within the range of the standard curves whereas serum samples for BDNF were diluted 1:75 to be within the range of the standard curves. In general, duplicate determinations of absorbancy with an intra-assay variance above 5% were determined again another day and the non-reliable measure discarded. The mean value of the duplicates was used in the statistical analyses.

### Genotyping

Single-nucleotide polymorphisms (SNPs) selected for genotyping were identified from HapMap data (http://www.hapmap.org, version 27 dbSNP 126 (NCBI build 36)) using the tagger selection algorithm in Haploview (pair-wise tagging function, *r*^2^ threshold=0.8).^32^ Genotyping was performed using the Sequenom MassARRAY platform. In total, 10 SNPs located within the gene region of the *SORT1* gene were selected for genotyping, however, one SNP failed, and one SNP was excluded from further analysis owing to a minor allele frequency below 0.005. Thus, after quality control, eight SNPs with nice cluster plots were left for analyses.

### Chronic alcohol treatment of mice

To determine the effect of alcohol treatment on sortilin levels in the hippocampus, a group of experimentally naive C57BL/6 J male mice (*n*=10, 12–16 weeks of age) was subjected to a 4-week forced drinking regimen. During the first week, animals were given a choice between water and an alcohol solution in water (2% for the first 3 days, then 5% for 4 days). Subsequently, the water bottle was removed and the mice were provided 5% alcohol in water as the sole drinking source for 3 days. This was then increased to 10% for 4 days and finally 20% for the remaining 2 weeks. At the end of the 4 weeks, the mice were killed and their hippocampi removed. The levels of sortilin were subsequently analysed by western blotting and compared with those of naive mice.

### Fluoxetine treatment of mice

For chronic antidepressant treatment, the experiments were carried out as described in Di Lieto *et* *al*.^33^ Three wild-type males aged 52 weeks were used in each group. The mice had free access to either tap water or tap water with 0.08 mg ml^−1^ addition of fluoxetine (Sigma-Aldrich #1279804) for 21 days. On the final day of treatment, the animals were euthanized by cervical dislocation and the hippocampus and prefrontal cortex samples were rapidly dissected. The samples were hereafter homogenized and lysed in TNE buffer.

### Statistical analyses

The software PLINK^34^ was used to perform quality control of the genotypes and to calculate the trend for association between the SNP alleles and depression.

Multiple linear regression models were used to assess independent determinants of the serum sortilin level, and testing was performed using likelihood-ratio statistics. Natural logarithm transformation was used to normalize the distribution of the sortilin levels. The analyses were conducted using Stata 13 (StataCorp, College Station, TX, USA) and a *P*-value of <0.05 was considered to be statistically significant. None of the analyses were corrected for multiple testing. Univariate linear regression analyses using socio-demographic variables, health indicators, lifestyle indicators and genotypes to assess independent variables of serum sortilin were performed. Likewise, we performed exploratory tests for some potentially relevant two-way interactions. We considered the interaction between the significant main effects and between the main effects and sex. Correlations between the serum measurements were calculated using the Spearman's rank correlation coefficient. Differences between sexes and between cases and controls in serum measurements, socio-demographic data, lifestyle indicators and health indicators were evaluated with the Wilcoxon two-sample rank-sum test for continuous variables and *χ*^2^ test for categorical variables.

## Results

The case group comprised 25 males and 127 females, and the control group comprised 46 males and 170 females. No significant differences in serum sortilin, BDNF or VEGF levels between sexes were observed (Table 1). Likewise, no significant differences were observed among males and females when analysing the cases and controls separately (not shown). A significantly higher mean serum sortilin level was observed in depressed individuals compared with controls (Table 1, Supplementary Figure 2). A further exploration of this showed significantly higher serum sortilin level in female cases compared with female controls. The same tendency was observed in male cases compared with male controls; however, the difference was not significant (not shown).

We previously reported significantly higher serum BDNF and VEGF levels in cases compared with controls.^25, 26^ However, the present study sample includes fewer individuals than the previous studies, and, for transparency, the medians and means for serum BDNF and VEGF are therefore presented in Table 1.

Additional sample characteristics are presented in Table 2. We observed a significant difference between cases and controls in hours of physical activity, smoking habits, previous depressive episodes, previous diagnoses with other mental disorders, antidepressant treatment and numbers of experienced stressful life events (Table 2). We also observed a significant difference between males and females in the mean age (*P*=0.005), BMI values (*P*=0.006) and alcohol consumption (*P*=0.03; Supplementary Table 1).

**Figure 1:**
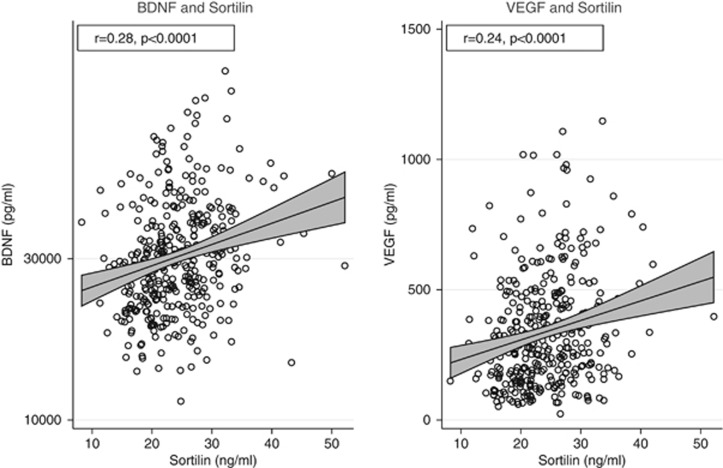
Scatter plots illustrating correlations between the serum sortilin level and serum levels of BDNF and VEGF, respectively. BDNF, brain-derived neurotrophic factor; VEGF, vascular endothelial growth factor.

Within individual, serum levels of sortilin were positively correlated with serum BDNF and VEGF (*r*=0.28, *P*<0.0001 and *r*=0.24, *P*<0.0001, respectively; Figure 1).

### Regression model

To understand which factors might influence the serum sortilin concentration, we performed univariate analyses of socio-demographic determinants, lifestyle indicators, health indicators and genotypes (Table 3). The regression analyses showed that the mean serum sortilin concentration increased significantly with increasing age, BMI, serum VEGF and serum BDNF levels and with high alcohol intake, depression and previous depressive episodes. However, no association between the serum sortilin level and previous depressive episodes was observed in the control group. The mean serum sortilin concentration decreased significantly in individuals with the CT genotype of rs11581665 compared with the CC genotype and in individuals with the AG genotype of rs3768497 compared with the GG genotype. No significant differences were observed between the individuals homozygous for the CC genotype compared with the TT genotype for rs11581665 and between individuals homozygous for the GG genotype compared with the AA genotype for rs3768497. Further exploration of these findings also showed that the significantly lower mean serum sortilin levels observed for heterozygous individuals were only observed in the control group. The mean serum sortilin levels depending on the rs11581665 and rs3768497 genotype are presented in the Supplementary Table 2. BDNF was the most significantly associated variable in the univariate analyses and explaining 5% of the variability of the serum sortilin level. The other significantly associated variables explained 1–5% of the variability each.

### Multiple regression model

To build a model of the serum sortilin level, we used a stepwise forward inclusion and backward elimination procedure, and the final model contained five significant determinants: depression, serum BDNF, serum VEGF, alcohol and BMI. None of the tested two-way interactions were statistically significant and thus not included in the final model. Regression coefficients from the final multiple regression model are shown in Table 4. The final regression model shows that compared with controls and adjusting for serum levels of BDNF and VEGF, BMI and alcohol intake, the mean sortilin level is significantly increased in depressed individuals. Compared with the individuals with an alcohol intake ⩽20 g per day and ⩽30 g per day for females and males, respectively, and adjusting for serum levels of BDNF and VEGF, BMI and depression, the mean sortilin levels are significantly increased in individuals with an alcohol intake above 20 and 30 g per day for females and males, respectively. In addition, the mean serum sortilin levels increases for every unit increase in serum BDNF, VEGF and BMI, respectively, when adjusting for the above-mentioned variables.

### Effect of alcohol and antidepressants on sortilin in the central nervous system

To further understand how factors associated with increased serum levels of soluble sortilin affects sortilin protein levels in the central nervous system (CNS), we conducted a series of mouse experiments. Exercise and diet have previously been reported to affect sortilin levels in the brain and liver, respectively.^35, 36, 37^ However, we here observed no effect of long-term alcohol drinking or administration of the antidepressant fluoxetine on sortilin protein levels in the mouse CNS (Supplementary Figure 3).

### Genetic analyses

None of the successfully genotyped SNPs showed evidence of association with depression in the single SNP analyses (Supplementary Table 3).

## Discussion

To the best of our knowledge, the present study comprising 368 individuals is so far the most comprehensive study of sortilin and depression. The relationship between depression and serum levels of sortilin and the association between depression and genetic markers located within the *SORT1* gene were investigated. Furthermore, the correlation between serum sortilin levels and serum BDNF and VEGF levels, respectively, were investigated. A secondary aim of our study was to identify potential determinants of serum sortilin levels and to build a multivariate model. Finally, we addressed whether high alcohol consumption and antidepressant treatment affects sortilin expression in the mouse CNS.

In summary, we observed a significant increase in serum sortilin level in depressed individuals compared with controls and significant correlations between serum levels of sortilin levels and the serum levels of BDNF and VEGF, respectively. None of the genotyped SNPs showed evidence of association with depression; however, the level of serum sortilin was significantly associated with two SNPs. The univariate regression analyses of factors influencing the serum sortilin level showed that the mean concentration of serum sortilin increased significantly with increasing age, BMI, serum VEGF and serum BDNF levels in addition to high alcohol intake and previous depressive episodes. Our final multiple regression model included five predictors: depression, serum BDNF, serum VEGF, alcohol intake and BMI. None of the analyses were corrected for multiple testing, however.

Only a few other studies have investigated serum sortilin levels in depression. In accordance with our study, Zhou *et al.* also found a significantly higher sortilin level in 40 depressed individuals compared with 50 controls.^19^ Interestingly, a recent study investigating differentially expressed proteins in 12 depressed individuals after electroconvulsive therapy observed a significant decrease in serum sortilin level following treatment.^38^

Interestingly, high sortilin levels was associated with mild and moderate depression, but not with severe depression. Likewise, no association between serum BDNF levels and severe depression or between serum VEGF levels and severe depression were observed in our previous studies of the same individuals.^25, 26^ Our study only included 26 individuals with a severe depression compared with 56 and 70 individuals with a mild and moderate depression, respectively. Our finding could thus be a result of low power to detect association in this subgroup of cases.

In the univariate analyses, we observed no association between serum sortilin levels and antidepressant treatment; however, only 48 individuals (16%) reported to receive antidepressant treatment, of which 43 were cases and five were controls. Sub-analyses in patients only, likewise, showed no significant differences in the serum sortilin level between depressed individuals receiving antidepressant treatment compared with those that did not receive treatment.

Our data demonstrate that apart from depression, several other factors may influence the serum sortilin level. Of note, both BDNF and VEGF display significant correlation with sortilin, suggesting that altered neurotrophic factor activity may affect sortilin expression and/or shedding or vice versa. Most studies investigating the serum BDNF levels (including our previous studies^25, 26^) have used commercial kits that do not differentiate mature BDNF from proBDNF. In future studies, it would be relevant to investigate whether mature BDNF and proBDNF are differentially correlated to sortilin.

The observed association between sortilin and BMI is supported by the fact that sortilin is also expressed in adipose tissue and in the liver, and is involved in lipid metabolism.^5^ Indeed, soluble sortilin in serum was previously found to correlate positively with PCSK9, a potent negative regulator of lipoprotein particle receptors.^18^ Interestingly, western-type diet and administration of saturated fatty acids resulted in posttranslational downregulation of hepatic sortilin without affecting mRNA levels,^35, 36^ while a recent study reported that 4 months of moderate treadmill running significantly reduced sortilin protein levels in the mouse cortex,^37^ altogether suggesting that a combination of diet and exercise may have marked and potentially opposite effects on the levels of circulating soluble sortilin and full-length sortilin in liver and brain, respectively. Likewise, we observed that a high intake of alcohol was associated with an increase in the mean sortilin concentration in serum although long-term alcohol administration had no effects on full-length sortilin protein levels in the mouse hippocampus. Interestingly, although still a matter of debate, BDNF has been hypothesized to be involved in ethanol–induced neurodegeneration in the adult brain, and high alcohol intake has been associated with lower serum BDNF levels.^39, 40^ Depression is furthermore known to predispose for alcohol abuse.^41^ As information on alcohol intake in the present study was obtained from the questionnaire, the risk of biased reporting is, however, increased. Potentially, several other biological and environmental factors not considered in this study might influence the serum sortilin level. Storage time of the samples and diurnal variation may, for example, affect the serum sortilin levels. Given the stable structure of sortilin,^5^ we have, however, no reason to believe that the generation of soluble sortilin varies dramatically on a daily and monthly basis, but future studies should address the half-life and stability of soluble sortilin in the circulation. Likewise, unpublished data indicate that sortilin levels are not affected to any major extent by variation in sampling and processing times or by multiple freeze–thaw cycles. However, future systematic studies on this issue are warranted.

The present study was limited by the data collected in the PRISME study and thus no power calculations were performed before initiation of the study.

None of the genotyped SNPs were associated with depression. This finding is supported by a large and comprehensive genetic study for depression including more than 18 000 individuals in the discovery phase and more than 57 000 individuals in the replication phase, found no evidence of association between depression and the chromosome region comprising *SORT1*.^42^ Interestingly, however, the intronic SNP, rs11581665, was significantly associated with serum sortilin levels. We observed a significantly higher serum sortilin level in individuals homozygous for the major allele (C-allele) compared with heterozygous individuals. Likewise, the mean serum sortilin level in individuals heterozygous for another intronic SNP, rs3768497, was significantly decreased compared with individuals homozygous for the major allele (G-allele). Analyses of the cases and controls separately (rs11581665 and rs3768497) revealed a significant difference in the serum sortilin level in the control group only. According to the NCBI eQTL Browser and the Genotype-Tissue Expression project, none of these SNPs are known eQTLs. Thus the relevance of these findings, if any, remains to be elucidated. Larger studies on serum sortilin levels and these genotypes are therefore warranted.

How increased levels of soluble sortilin in serum contribute to the pathophysiology of depression still remains elusive. Studies in rats have shown that sortilin mRNA was two-fold reduced in the hippocampus of rats resistant to the experimental depression paradigm learned helplessness compared with non-resistant rats,^43^ indicating a potential correlation between sortilin expression in the CNS and susceptibility to depression. Of note, our present studies show that antidepressant treatment itself did not alter sortilin protein levels in the hippocampus or cortex of mice. Hence, the increased soluble sortilin in serum of depressed patients may mirror abnormal CNS expression as a consequence of depression itself but not treatment. Furthermore, abnormal amounts of soluble sortilin likely perturb several neurotrophic factor systems. Indeed, soluble sortilin has been suggested to function as a decoy receptor for progranulin, thereby promoting its neurotrophic activity.^44^ On the other hand, soluble sortilin inhibits proteolysis of proBDNF and the generation of mature BDNF.^12^

## Conclusion

In conclusion, our results suggest a role of circulating sortilin in depression and that the serum sortilin level is influenced by several factors, including neurotrophic factor activity. Owing to the large amount of information, this is, to the best of our knowledge, the most comprehensive study of sortilin in depression. However, further investigations are needed to replicate our findings. Studies including additional factors that potentially could influence the sortilin level in addition to longitudinal studies of sortilin in depression are also warranted.

## Figures and Tables

**Table 1:** Medians and means for sortilin, BDNF and VEGF

	*Median (95 % CI)*		*Mean (std)*		P-value*
	Male (*n*=71)	Female (*n*=297)	Male (*n*=71)	Female (*n*=297)	
Sortilin (ng ml^−1^)	24 (17–36)	24 (15–34)	25 (6)	24 (6)	0.33
					
	Male (*n*=70)	Female (*n*=296)	Male (*n*=70)	Female (*n*=296)	
BDNF (pg ml^−1^)	29 972 (20 819–43 312)	29 717 (19 742–42 980)	30 379 (6644)	30 267 (7148)	0.83
					
	Male (*n*=69)	Female (*n*=286)	Male (*n*=69)	Female (*n*=286)	
VEGF (pg ml^−1^)	259 (76–720)	310 (87–735)	324 (239)	344 (207)	0.19
					
	Case (*n*=152)	Control (*n*=216)	Case (*n*=152)	Control (*n*=216)	
Sortilin (ng ml^−1^)	26 (17–40)	23 (15–33)	26 (7)	23 (6)	0.0002
					
	Case (*n*=151)	Control (*n*=215)	Case (*n*=151)	Control (*n*=215)	
BDNF (pg ml^−1^)	30 817 (21 088–44 732)	29 225 (19 291–41 417)	31 228 (7388)	29 628 (6735)	0.04
					
	Case (*n*=150)	Control (*n*=205)	Case (*n*=150)	Control (*n*=205)	
VEGF (pg ml^−1^)	361 (107–872)	245 (75–661)	407 (230)	290 (186)	<0.0001

Abbreviations: BDNF, brain-derived neurotrophic factor; CI, confidence interval; VEGF, vascular endothelial growth factor.

**Table 2:** Sample characteristics (*n*=368)

*Sociodemographics*	*Details*			*Statistics*
	*Case*	*Control*	*Total*	P*-value*
Male (%)	25 (16)	46 (21)		
Female (%)	127 (84)	170 (79)		
Mean age in years (s.d.)	47.1 (9.4)	46.3 (10.2)	46.7 (9.8)	0.55^a^

*Lifestyle indicators*				
Physical activity (**70**)				0.022^b^
>4 h per week (%)	52 (41.3)	94 (54.6)	146 (49.0)	
⩽4 h per week (%)	74 (58.7)	78 (45.4)	152 (51.0)	
Smoking (**1**)				0.011^b^
Smoking (%)	41 (27)	32 (14.9)	73 (19.9)	
Previous smoker (%)	53 (34.9)	77 (35.8)	130 (35.4)	
Never smoker (%)	58 (38.1)	106 (49.3)	164 (44.7)	
BMI (**2**) kg/m^2^ (s.d.)	26.0 (5.4)	25.3 (4.3)	25.6 (4.8)	0.40^a^
Alcohol (**3**)				0.131^b^
⩽20/30 g per day (%)	138 (90.8)	202 (94.8)	340 (93.2)	
>20/30 g per day (%)	14 (9.2)	11 (5.2)	25 (6.8)	

*Health indicators*				
*Depression*				
Mild depression (%)	56 (36.8)			
Moderate depression (%)	70 (46.1)			
Severe depression (%)	26 (17.1)			
Previous depressive episodes (**11**)				<0.001^b^
Previous depressive episodes (%)	79 (54.9)	26 (12.2)	105 (29.4)	
No previous depressive episodes (%)	65 (45.1)	187 (87.8)	252 (70.6)	
Previous diagnosis with other mental disorders (**26**)				<0.001^b^
Previous diagnosis with other mental disorders (%)	14 (10.7)	1 (0.5)	15 (4.4)	
No previous diagnosis with other mental disorders (%)	117 (89.3)	210 (99.5)	327 (95.6)	
Antidepressant treatment (**66**)				<0.001^b^
No antidepressant treatment (%)	102 (70.3)	152 (96.8)	254 (84.1)	
Antidepressant treatment (%)	43 (29.7)	5 (3.2)	48 (15.9)	
SLEs				<0.001^b^
No experienced SLEs	63 (41.5)	135 (62.5)	198 (53.8)	
One experienced SLE	42 (27.6)	58 (26.9)	100 (27.2)	
Two or more experienced SLEs	47 (30.9)	23 (10.6)	70 (19.0)	

Abbreviations: BMI, body mass index; SLE, stressful life event.

a Wilcoxon two-sample rank-sum test (Mann–Whitney test).

b Chi square test.

Number of missing individuals are listed in bold parenthesis in the first column.

**Table 3:** Univariate analyses

*Sociodemographics and lifestyle*	*Details*	*Linear regression*		
		*Coefficient*	*Test*	P*-value*
Male		0.04 (−0.03;0.11)	1.18	0.24
Age		0.004 (0.001;0.006)	2.78	**0.006**
Alcohol intake	>20 g per day for females and >30 g per day for males	0.13 (0.02;0.24)	2.41	**0.02**
Smoking	Present smoker	0.04 (−0.03;0.11)	1.02	0.31
	Previous smoker	0.004 (−0.06;0.06)	0.13	0.9
BMI		0.01 (0.003;0.01)	2.98	**0.003**
Physical activity	>4 h per week	−0.06 (−0.12;0.003)	−1.88	0.061
One experienced SLEs		−0.01 (−0.07;0.05)	−0.26	0.8
Two or more experienced SLEs		−0.04 (−0.11;0.03)	−1.05	0.29
				
*Health indicators*				
Depression		0.10 (0.05;0.16)	3.84	**<0.001**
	Mild	0.10 (0.03;0.18)	2.73	**0.007**
	Moderate	0.14 (0.08;0.21)	4.13	**<0.001**
	Severe	−0.004 (−0.11;0.10)	−0.08	0.93
Previous depressive episodes		0.06 (0.001;0.12)	2.00	**0.05**
Previous diagnosed with other mental disorders		−0.01 (−0.15;0.13)	−0.16	0.88
Antidepressant treatment		0.07 (−0.01;0.15)	1.67	0.1
				
*Serum measurements*				
Serum VEGF		2e−4 (1e−4;4e−4)	4.21	**<0.001**
Serum BDNF		9e−6 (5e−6;1e−5)	4.64	**<0.001**
				
*Genotypes*				
rs11581665	CT genotype	−0.09 (−0.16;−0.03)	−2.84	**0.005**
	TT genotype	−0.02 (−0.19;0.14)	−0.27	0.79
rs12037569	GT genotype	0.02 (−0.05;0.08)	0.49	0.63
	TT genotype	0.08 (−0.10;0.27)	0.9	0.37
rs17585355	CA genotype	0.01 (−0.07;0.10)	0.34	0.74
	CC genotype	0.10 (−0.26;0.47)	0.57	0.57
rs17646665	GA genotype	0.04 (−0.03;0.11)	1.07	0.29
	GG genotype	0.31 (−0.20;0.82)	1.18	0.24
rs3768497	AA genotype	−0.05 (−0.14;0.04)	−1.07	0.29
	AG genotype	−0.06 (−0.12;−0.007)	−2.22	**0.027**
rs413582	CT genotype	−0.01 (−0.08;0.05)	−0.39	0.7
	TT genotype	0.01 (−0.07;0.09)	0.26	0.8
rs464218	CC genotype	−0.04 (−0.12;0.04)	−0.97	0.33
	CT genotype	−0.03 (−0.09;0.03)	−0.9	0.37
rs7536292	CC genotype	0.11 (−0.05;0.28)	1.35	0.18
	CT genotype	−0.0005 (−0.06;0.06)	−0.02	0.99
rs6265 (val66met)	AA genotype	0.006 (−0.11;0.12)	0.1	0.92
	GA genotype	0.05 (−0.01;0.10)	1.55	0.12

Abbreviations: BDNF, brain-derived neurotrophic factor; BMI, body mass index; SLE, stressful life event; VEGF, vascular endothelial growth factor.

*P*-values for significantly associated variables are shown in bold.

**Table 4:** Multiple regression—the final model (*n*=349)

	*Coefficient (95% CI)*	*s.e.*	*Test*	P*-value*
Depression	0.071 (0.017;0.13)	0.028	2.6	0.01
Serum BDNF	7.3e−6 (3.6e−6;1.1e−5)	1.9e−06	3.8	<0.001
Serum VEGF	1.5e−4 (2.2e−5;2.8e−4)	6.6e−05	2.3	0.022
Alcohol	0.14 (0.038;0.25)	0.053	2.7	0.007
BMI	0.0071 (0.0016;0.013)	0.0028	2.6	0.011
Constant	2.7 (2.5;2.8)	0.087	31	<0.001

Abbreviations: BDNF, brain-derived neurotrophic factor; BMI, body mass index; CI, confidence interval; VEGF, vascular endothelial growth factor.
