# Supplementary figures and images for: Increased serum levels of sortilin are associated with depression and correlated with BDNF and VEGF
Source: Transl Psychiatry. 2015 Nov 10;5(11):e677–. doi: 10.1038/tp.2015.167 (PMC5068760; doi:10.1038/tp.2015.167)

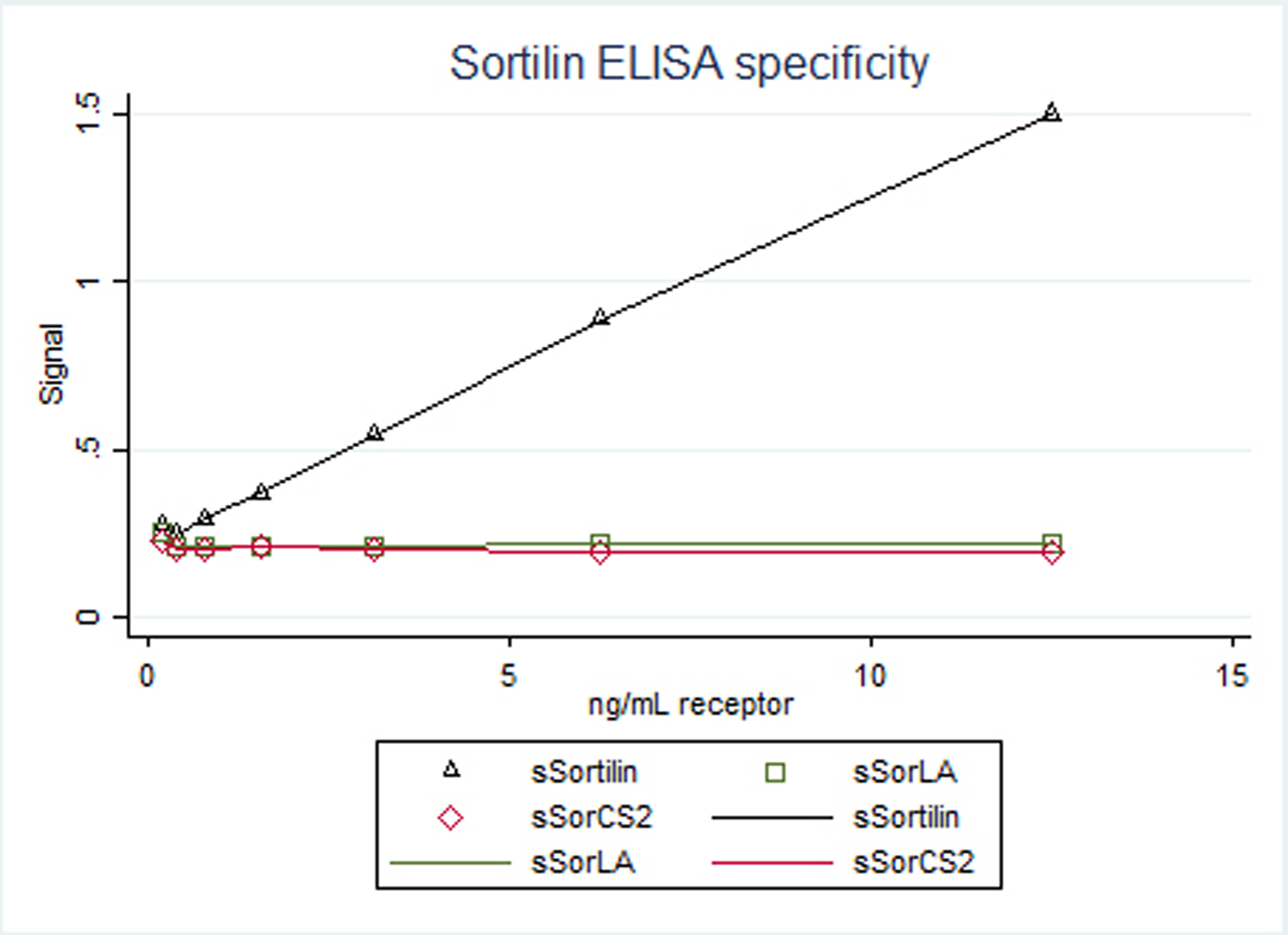

Supplement: Supplementary Figure 1 [file tp2015167x1.tif]

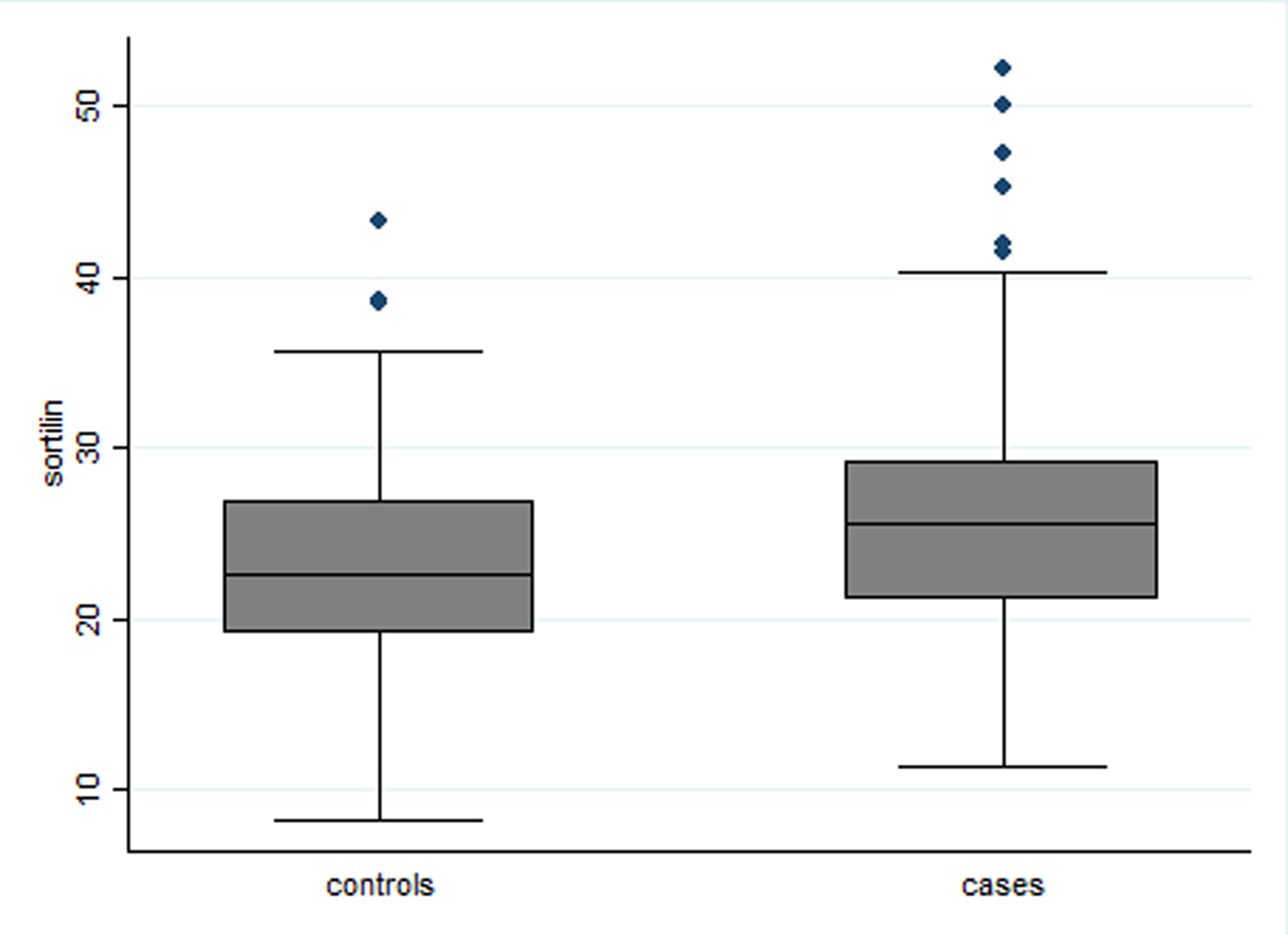

Supplement: Supplementary Figure 2 [file tp2015167x2.tif]

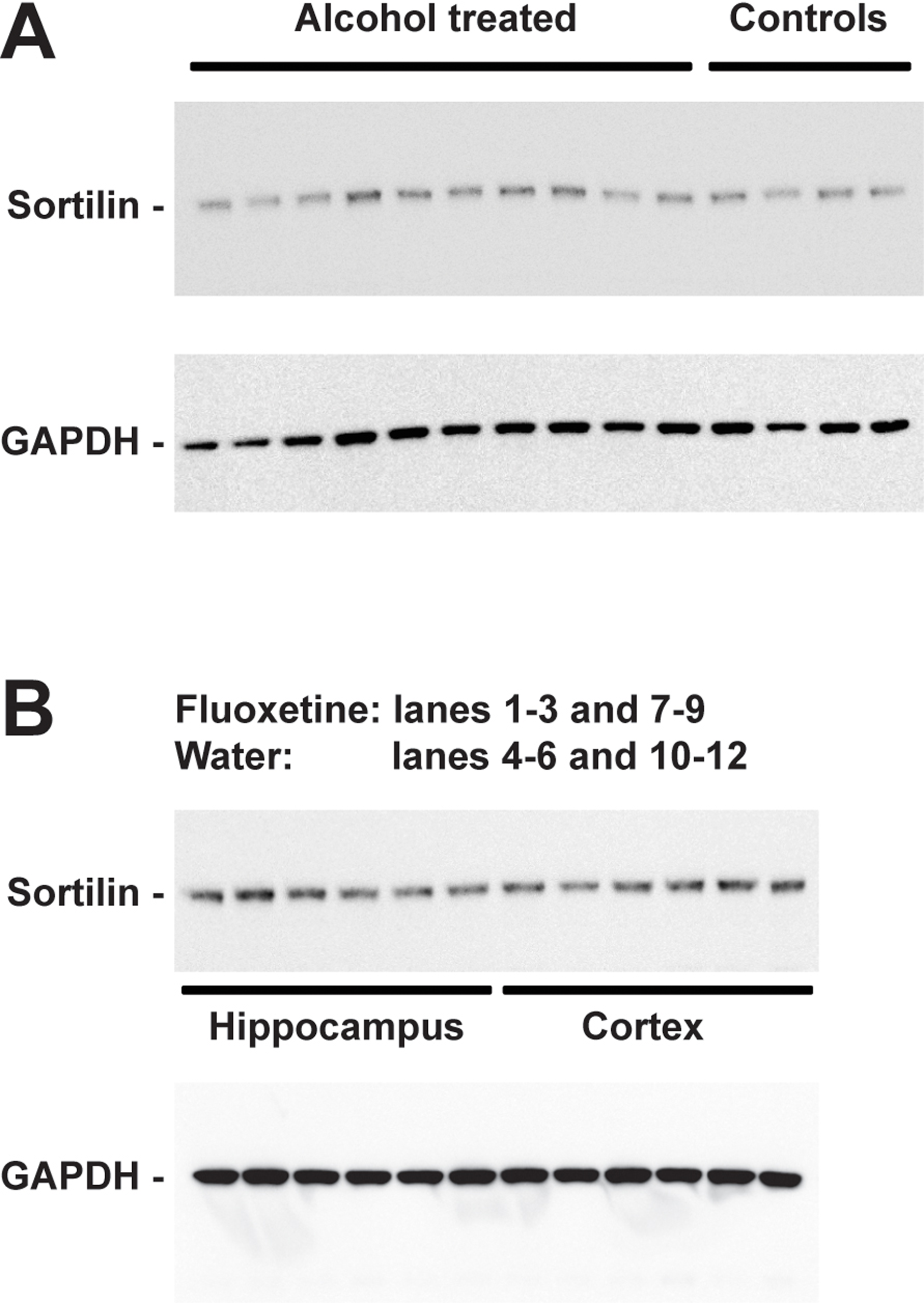

Supplement: Supplementary Figure 3 [file tp2015167x3.tif]
