# Supplementary material for: Increased serum levels of sortilin are associated with depression and correlated with BDNF and VEGF
Source: Transl Psychiatry. 2015 Nov 10;5(11):e677–. doi: 10.1038/tp.2015.167 (PMC5068760; doi:10.1038/tp.2015.167)
Supplement: Supplementary Information [file tp2015167x7.doc]

**Supplementary figure 1 Legend**

Specificity and sensitivity of sortilin ELISA

The sortilin ELISA was performed as described in the methods section using a concentration gradient of purified recombinant extracellular domains of human sortilin, SorCS2, and SorLA as samples. As evident from the graph, the ELISA exclusively detects human sortilin and not the related receptors SorCS2 and SorLA.

**Supplementary figure 2 Legend**

Box plots illustrating significantly higher serum sortilin levels in depressed cases compared to control individuals

**Supplementary figure 3 Legend**

Sortilin levels in mice following alcohol or antidepressant administration.

(A) Western blotting showing sortilin levels in the hippocampus of mice (n=10) subjected to a four-week forced alcohol drinking regimen compared to experimentally naïve mice. Western blotting for GAPDH served as loading control.

(B) Sortilin and GAPDH levels in the hippocampus and cortex of mice administered water containing fluoxetine or water alone for 21 days. No significant difference in sortilin levels was observed in any of the conditions. 50 µg protein was loaded in each lane.
